# Littoral sediment arsenic concentrations predict arsenic trophic transfer and human health risk in contaminated lakes

**DOI:** 10.1371/journal.pone.0293214

**Published:** 2023-10-19

**Authors:** Erin A. Hull, Rebekah R. Stiling, Marco Barajas, Rebecca B. Neumann, Julian D. Olden, James E. Gawel

**Affiliations:** 1 Environmental Sciences, School of Interdisciplinary Arts and Sciences, University of Washington Tacoma, Tacoma, Washington, United States of America; 2 Water and Land Resources Division, King County Department of Natural Resources and Parks, Seattle, Washington, United States of America; 3 Department of Civil and Environmental Engineering, University of Washington, Seattle, Washington, United States of America; 4 School of Aquatic and Fishery Sciences, University of Washington, Seattle, Washington, United States of America; VIT University, INDIA

## Abstract

Lake sediments store metal contaminants from historic pesticide and herbicide use and mining operations. Historical regional smelter operations in the Puget Sound lowlands have resulted in arsenic concentrations exceeding 200 μg As g^-1^ in urban lake sediments. Prior research has elucidated how sediment oxygen demand, warmer sediment temperatures, and alternating stratification and convective mixing in shallow lakes results in higher concentrations of arsenic in aquatic organisms when compared to deeper, seasonally stratified lakes with similar levels of arsenic pollution in profundal sediments. In this study we examine the trophic pathways for arsenic transfer through the aquatic food web of urban lakes in the Puget Sound lowlands, measuring C and N isotopes–to determine resource usage and trophic level–and total and inorganic arsenic in primary producers and primary and secondary consumers. Our results show higher levels of arsenic in periphyton than in other primary producers, and higher concentrations in snails than zooplankton or insect macroinvertebrates. In shallow lakes arsenic concentrations in littoral sediment are similar to deep profundal sediments due to arsenic remobilization, mixing, and redeposition, resulting in direct arsenic exposure to littoral benthic organisms such as periphyton and snails. The influence of littoral sediment on determining arsenic trophic transfer is evidenced by our results which show significant correlations between total arsenic in littoral sediment and total arsenic in periphyton, phytoplankton, zooplankton, snails, and fish across multiple lakes. We also found a consistent relationship between percent inorganic arsenic and trophic level (determined by δ^15^N) in lakes with different depths and mixing regimes. Cumulatively, these results combine to provide a strong empirical relationship between littoral sediment arsenic levels and inorganic arsenic in edible species that can be used to screen lakes for potential human health risk using an easy, inexpensive sampling and analysis method.

## Introduction

Arsenic contamination of aquatic ecosystems has well established negative impacts on environmental and human health [1, 2]. Anthropogenic activities such as mining, herbicide applications, groundwater pumping, and smelting have led to increased arsenic exposure globally [3–6]. Widely known as a neurotoxin and carcinogen, exposure to arsenic can adversely affect virtually every system in the human body [7]. Long-term exposure at low levels can lead to co-morbidities such as diabetes, hypertension, cardiovascular diseases, and decreased fertility [8–11]. Environmental toxicity studies have also documented far-ranging effects of arsenic exposure on variety of species including oxidative damage in goldfish [12], immobility in New Zealand mud snails [13], memory loss [14] and diminished neurogenesis in zebrafish [15], and reduced reproduction in aquatic midge insects [16].

Arsenic has been found to transfer through food webs from one trophic level to the next [17, 18]. Total arsenic concentrations biodiminish with increasing trophic level in most aquatic food chains [19], as does the proportion of arsenic that is inorganic (iAs) [20]. iAs chemical species are more toxic to humans and other biota compared to organic (oAs) species [21, 22]. Thus, differences in toxicity between inorganic and organic forms of arsenic have increased attention on As speciation, especially as an important tool in the assessment of potential health risks [23–25]. In contaminated aquatic systems, primary producers and their direct consumers typically contain higher concentrations of total As and higher percentages of iAs species [26] compared to organisms that feed higher on the food chain, such as fishes [27].

In the Puget Sound lowland region of Washington State, USA, widespread legacy arsenic pollution is primarily attributable to historical smelting operations. The American Smelting and Refining Company (ASARCO) operated a copper smelter in the City of Ruston from 1890 until 1986; its emissions stack was once the tallest in the world. For almost a century, the smelter produced a large plume depositing arsenic and lead over the landscape, including the regions’ lakes. Extensive mapping of the contamination in the region’s terrestrial environment has occurred, along with intense topsoil remediation efforts after the smelter was deemed a Superfund site in 1987 [28]. Previous research has revealed persistence of this legacy arsenic pollution in the sediments of lakes within the zone of deposition; some containing concentrations over 200 μg As g^-1^ in the deep profundal zone of these lakes [5]. Further investigations have shown the sediment arsenic moves into lake water and is bioaccumulated in the lake food web [27, 29, 30].

Evidence suggests that arsenic bioaccumulation is greater in shallow lakes compared to deeper lakes with the same concentration of arsenic in profundal sediments [27, 29]. In deeper lakes that maintain seasonal stratification, remobilized iAs in the hypolimnion remains separate from oxygenated surface waters, where most oxygen-requiring organisms reside during the majority of their life cycles, reducing biotic exposure. By contrast, shallower lakes experience periodic mixing events throughout the warmer months (polymictic), resulting in dissolved iAs being remobilized from sediment into oxygenated surface waters and nearshore littoral areas during brief periods of stratification and hypolimnetic anoxia [29, 31]. This situation results in significant spatial overlap between dissolved iAs and oxic habitat in shallow lakes [29]. Furthermore, sediment arsenic flux estimates detailed in Barrett et al. [30] imply an enhanced renewal of arsenic from profundal sediments to littoral zones occurs in shallow lakes, compared to deeper lakes where steeper basin morphometry and strong summertime thermal stratification may limit arsenic redistribution from deep-to-shallow-water sediments [32]. Recent research also indicates that the relationship between arsenic concentrations in aquatic organisms and littoral media (i.e., sediment and water) is stronger than the relationship between arsenic in organisms and hypolimnetic arsenic concentrations [27]. In the study by Hull et al. [27], modeled consumption of edible tissues of fish from two lakes, both containing over 200 μg g^-1^ of arsenic in profundal sediments, resulted in an elevated cancer risk (> 10^−5^) exceeding Washington State health standards in the shallow lake, but not for the deeper, seasonally stratified lake. Thus, it appears that arsenic-contaminated shallow lakes pose a particularly problematic combination of physical, chemical, and biotic factors that result in greater arsenic bioavailability.

In the present study we investigate potential differences in trophic transfer pathways for arsenic between lakes with contrasting morphometry and mixing regime, and explore ways to predict iAs concentrations in edible aquatic animals harvested from arsenic contaminated lakes. We hypothesize that the potential health risk from consuming animals (determined by the amount of iAs in edible tissues) will be predicted by littoral sediment arsenic concentrations and trophic position in the food web, but otherwise will not vary due to differing mixing regime or maximum lake depth. To explore our hypothesis, we measured As in littoral sediment, oxygenated waters, periphyton, phytoplankton, macrophytes, zooplankton, insect larvae (*Chironomidae*), snails (*Bellamya chinensis*), and 2 species of sunfish (*Lepomis gibbosus and L*. *macrochirus*) collected from 10 study lakes in the Puget Sound lowland region of Washington State, USA. Lakes sampled range in arsenic contamination, lake morphometry, and mixing regime. There have been numerous studies shedding light on the trophodynamics of arsenic in freshwater food webs [17–19, 33–36] and comparing As uptake in organisms among sites with varying arsenic contamination [26, 27, 29, 37–40]. However, we are not aware of any studies to date that have compared arsenic trophic transfer across multiple lakes nor investigated littoral sediment arsenic content as a predictor for extent of arsenic uptake by the aquatic food web. Based on our results, we suggest measuring littoral sediment As provides a low-cost, accessible method for estimating the degree of risk associated with consuming aquatic species from arsenic-contaminated lakes.

## Materials and methods

### Study area and site characterization

**Fig 1:**
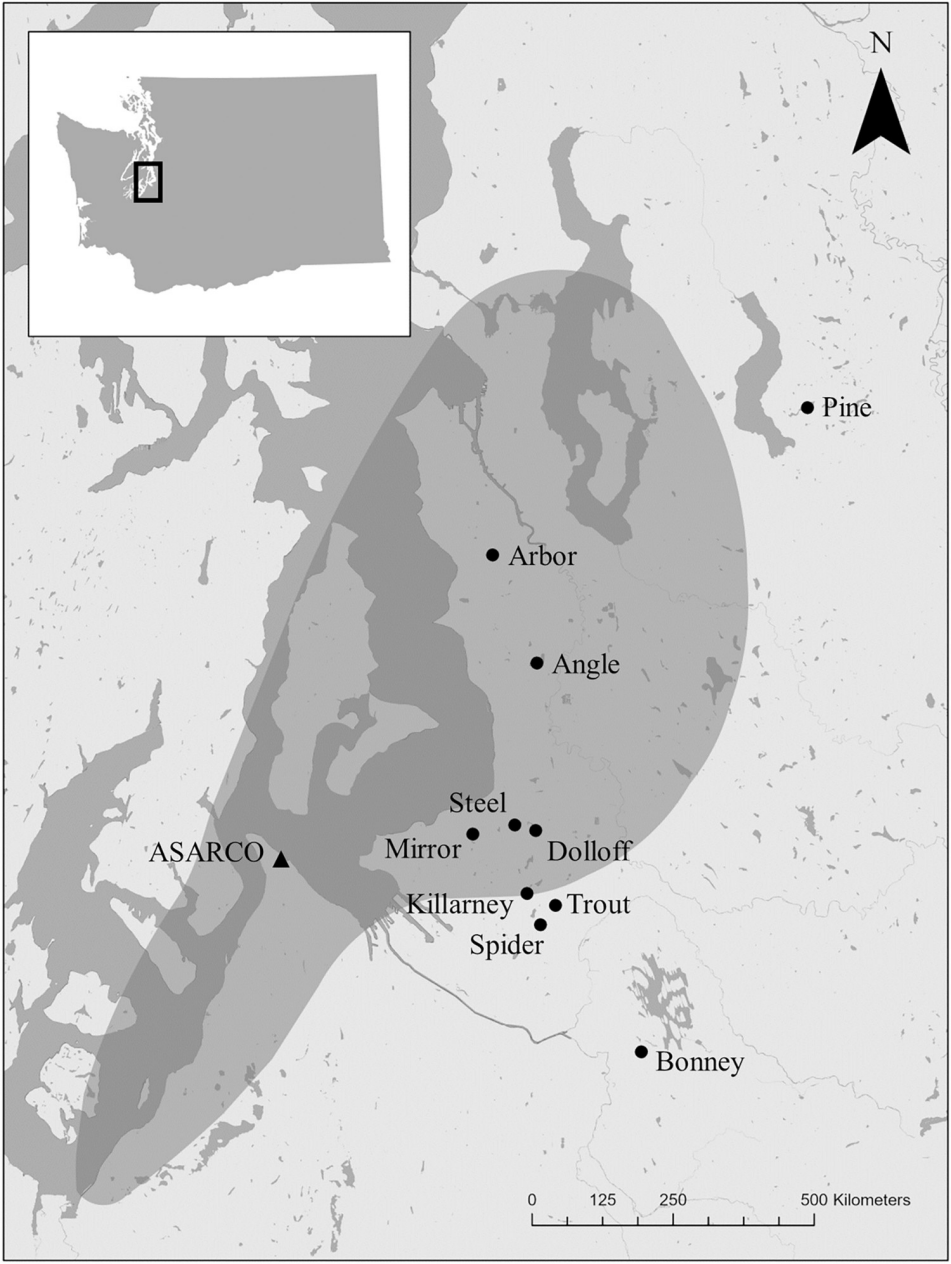
Map of study lakes and ASARCO smelter. The shaded area represents the estimated deposition zone of As from the smelter’s plume [5]. Open data provided by the Washington State Department of Ecology (WA State Boundary, Tacoma Smelter Plume Footprint, DNR Hydrography—Water Bodies—Forest Practices Regulation; https://geo.wa.gov/search?collection=Dataset).

Of the 10 study lakes located in the Puget Sound lowland region of Washington State, USA, 8 lakes are within the predicted deposition field of arsenic-contaminated smelter emissions ([28]; Fig 1), and two control lakes are located outside the predicted deposition field (Pine and Bonney Lakes). Of the 8 lakes potentially impacted by aerial fallout of arsenic, littoral sediment concentrations range from 7 to 213 μg As g^-1^ (Table 1).

**Table 1:** Morphometric characteristics and sediment As concentrations of study lakes.

Lake	latitude	longitude	lake area (km^2^)	max depth (m)	μg As L^-1^	n	μg As g^-1^	n
					oxic water column		littoral sediment	
Angle	47.427	-122.287	0.42	15.8	1.47 ± 0.12	3	35 ± 3.8	3
Arbor	47.493	-122.329	0.02	3.6			12	1
Bonney	47.189	-122.186	0.07	5.8	0.03 ± 0.04	3	10 ± 0.3	2
Dolloff	47.324	-122.285	0.08	4.8			20	1
Killarney	47.285	-122.292	0.12	4.6	20.42 ± 4.44	5	213 ± 83.5	5
Mirror	47.321	-122.342	0.08	6.6			19	1
Pine	47.587	-122.045	0.36	11.9			9	1
Spider	47.278	-122.266	0.01	2.3			22	1
Steel	47.327	-122.304	0.19	7.3	1.75 ± 0.35	3	50 ± 26.5	6
Trout	47.266	-122.279	0.07	7.9			7	1

Previous work reported that Angle Lake and Lake Killarney have the greatest concentrations of arsenic in profundal sediment for the 8 sampled lakes in the predicted deposition field (208 μg g^−1^ and 206 μg g^−1^, respectively; Table 1; [5]). Lake Killarney is shallow (maximum depth 4.6 m) and experiences frequent, year-round mixing events (termed ‘polymictic’). Angle Lake is deeper (maximum depth 15.8 m) and displays strong seasonal stratification [29]. Each type of organism described below was collected from Angle Lake and Lake Killarney, and a subset of those organisms was sampled from the 8 remaining lakes. A summary of samples collected from each lake and the analyses performed is provided in S1 Table.

### Sample collection

#### Sediment

Arsenic concentrations for profundal sediments were previously reported in Gawel et al. [5]. Littoral sediment samples were collected in 2018, 2019, and 2021 from the littoral zone of each lake. One to five samples were collected in the littoral zone (<2m depth) of each lake using a dredge (Wildco stainless steel petite ponar). In the laboratory, samples were dried at 60°C for 72 h and homogenized using a porcelain mortar and pestle.

#### Water and food web sampling

Water samples for dissolved arsenic, and phytoplankton and zooplankton tows, were collected from a boat at approximately the deepest point in each lake as described previously [29]. In June 2019, water samples were collected using a peristaltic pump, filtered (0.45 μm Geotech cartridge filter), acidified with 1% HNO_3_ (v/v) in the laboratory, and allowed to stand for 14 days prior to analysis for dissolved arsenic. Duplicate phyto- and zooplankton samples were collected in 2016, 2017, and 2019 using a vertical net tow (20 μm and 153 μm mesh, respectively) from 1–2 m above the lakebed. Phytoplankton were then pre-filtered through a 153 μm sieve to remove zooplankton. Plankton were collected on 5.0 μm polycarbonate filters and either dried at 60°C (for total As) or stored at -80°C (for As speciation).

Periphyton was grown *in situ* on multiple 23 cm^2^ acrylic plates assembled into 3 x 3 plate frames and placed at 0.5 m depth in the approximate center of each lake. Plates were deployed for at least 35 days during the summers of 2019 and 2021. Both sides of each plate were scraped of periphyton with a plastic razor blade and rinsed with ultrapure reverse osmosis de-ionized water into a filtration vessel. Material was captured on 5.0 μm polycarbonate filters and subsamples were either dried at 60°C overnight or stored at -80°C. Triplicate grab samples of mixed submerged macrophytes were harvested from nearshore (1–2 m depth) using an aquatic plant rake (Pond & Beach Rake Gen 2) in August and September 2020 and cleaned of attached sediment. Roots were removed and random portions of each rake harvest were either dried at 60°C overnight or stored at -80°C.

Insect midge larvae (*Chironomidae*) were collected from sediments in July and August 2020 at multiple sites in each lake using a dredge (Wildco stainless steel petite ponar). Dredge samples were sifted through a mesh sieve to reveal larvae within. Larvae were transferred into a container of lake water on ice. In the laboratory, larvae were separated into groups of 9–10 individuals per composite sample and either dried at 60°C overnight or stored at -80°C. Chinese mystery snails (*Bellamya chinensis*) were collected in June 2019 and July 2021 from the littoral zone (< 2 m depth) of 6 lakes (S1 Table). Collection involved hand netting via snorkeling or from a boat. *B*. *chinensis* were placed on ice in sealed plastic bags on the boat, euthanized in the laboratory, then later thawed and body tissue removed from the shell. Pumpkinseed (*Lepomis gibbosus*) and bluegill sunfish (*L*. *macrochirus*) were collected by beach seining from 5 lakes (S1 Table) in June 2019 and 2021. A scientific collection permit was approved for fish by Washington Department of Fish and Wildlife (SCP Olden 22–172); no permit is required for non-vertebrate sample collection. Field site access does not require a permit for publicly accessible lakes in Washington. Fish were euthanized and muscle tissue dissected. Fish and snail tissues were either dried at 60°C overnight or stored at -80°C.

### Laboratory analyses

Oven-dried samples of periphyton, phyto- and zooplankton, macrophytes, *Chironomidae*, snail whole soft tissue, and fish tissue were divided into 2 subsamples for total arsenic and δ^13^C and δ^15^N analysis. Small portions (< 3 mg) of each food web constituent were transferred into tin capsules for stable isotope analysis. All samples stored at -80°C were prepared for arsenic speciation by drying at 85°C. Macrophyte, snail, and fish tissue samples were homogenized using a porcelain mortar and pestle, while all other samples were used in entirety for arsenic analysis.

#### Total arsenic

Samples for total arsenic analysis were prepared by microwave-assisted (CEM MARS 5) total digestion protocol (modified EPA method 3015a) using trace metal grade HNO_3_ in pressurized digestion vessels. After digestion, sample solutions were diluted to 2% (v/v) HNO_3_. Concentrations of total arsenic in water and digested sediment, periphyton, plankton, *Chironomidae*, and *B*. *chinensis* and fish tissue samples were determined by inductively-coupled plasma mass spectrometry (ICP-MS; Agilent 7900). Calibration was performed using a certified multi-element standard (Agilent Multi-element calibration standard-2A). Efficacy of the digestion procedure was verified using certified reference material BCR-414 (Trace elements in plankton), NIST 2711a (Montana Soil II), and DOLT-5 (dogfish liver) which yielded a recovery of 90 ± 15% (n = 8), 92 ± 16% (n = 10) and 91 ± 12% (n = 22), respectively. Analytical accuracy of the ICP-MS method was assessed using certified reference material NIST 1640a (trace elements in natural water), which had a recovery of 87 ± 6% (n = 14) for arsenic. The limit of detection (LOD) for arsenic was 0.25 μg L^-1^. Samples below the limit of detection were assigned a value of 0.125 μg L^-1^ (half the LOD) [41].

#### Arsenic speciation

Arsenic speciation of phyto- and zooplankton collected in 2016 and 2017 was determined in the Trace Element Analysis laboratory at Dartmouth College using methods based on Taylor and Jackson [42]. Plankton samples were freeze-dried and transferred from polycarbonate filters into sample vials and 10% methanol was added. Samples were sonicated in a 30°C bath for 2 h, then filtered (0.2 μm) to remove residual solids. The supernatant was eluted with 20 mM (NH_4_)_2_CO_3_ (1.1 mL min^−1^) on an Agilent LC1120 liquid chromatograph. Arsenic species were separated using an anion-exchange column (Hamilton PRP-X100) at 35°C, and then analyzed on an Agilent 8900 triple quadrupole ICP-MS. Analytical accuracy was verified using a secondary standard with a recovery of 104 ± 2% (n = 3). The extraction efficiency (sum of species / total As by ICP-MS) for phytoplankton was 10 ± 6% (n = 4) and 23 ± 2% (n = 2) for zooplankton.

The speciation of arsenic in phyto- and zooplankton collected in 2019, periphyton, macrophytes, *Chironomidae*, *B*. *chinensis*, and fish tissue were also determined at the Trace Element Analysis laboratory at Dartmouth College, following a dilute acid heat assisted extraction (modified from [43]). An aliquot of the extract was diluted 1:1 with 200 mM NH_4_CO_3_ and analyzed for arsenic species by ion chromatography coupled to inductively coupled plasma mass spectrometry (IC-ICP-MS). A Dionex AS14 anion-exchange column was used with an NH_4_CO_3_ gradient to separate arsenate, arsenite, dimethylarsinic acid, monomethylarsonic acid, and arsenosugars (run time of 7 minutes, flow rate of 1 mL min^-1^, and injection volume of 20 μL). The system was calibrated using primary standard solutions of the arsenic species (Sigma Aldrich, Spex certiprep) and NIST 2669a urine level II was used for quality control. The extraction efficiency was 80 ± 16% (n = 6) for fish, 57 ± 27% (n = 6) for *B*. *chinensis*, 88 ± 9% (n = 2) for *Chironomidae*, 55 ± 14% (n = 2) for macrophytes, 124 ± 45% (n = 2) for zooplankton, 87 ± 0.9% for phytoplankton (n = 2), and 60 ± 17% (n = 2) for periphyton. Other studies report similar ranges for extraction efficiencies across different environmental materials using equivalent methods [42, 44, 45].

#### Stable isotope analysis

To explore how differences in lake morphometry may influence arsenic trophic transfer, we conducted a food-web investigation in lakes Angle and Killarney, two spatially proximate lakes with similar levels of arsenic in profundal surface sediments but differing maximum depths and mixing regimes. We used ^13^C/^12^C as a proxy for basal C resources and ^15^N/^14^N as a proxy for trophic position, which together provide an indication of resource use. Periphyton, plankton, macrophyte, *Chironomidae*, *B*. *chinensis*, and fish tissue samples were dried for 24–48 h at 60°C, homogenized with mortar and pestle, and encapsulated in tin capsules. Tissues were sent to University of California Davis Stable Isotope Facility and analyzed for ratios of stable isotopes (^13^C/^12^C and ^15^N/^14^N) using an elemental analyzer (PDZ Europa ANCA-GSL) interfaced to an isotope ratio mass spectrometer (PDZ Europa 20–20; Sercon Ltd., Cheshire, UK). Data are reported as permil (‰) relative differences from standards of Vienna Pee Dee Belemnite for C and atmospheric N, expressed as delta (δ) units. Long-term standard deviations for estimates of natural abundance stable isotope values based on reference material at University of California-Davis are 0.2‰ for δ^13^C and 0.3‰ for δ^15^N.

### Statistical methods

By leveraging arsenic speciation and stable nitrogen isotope analyses, we examined the relationship between the proportion of iAs and trophic position in each food web constituent from Killarney and Angle lakes. We fit a linear regression comparing the mean δ^15^N value (representing trophic position) to the mean iAs concentration for all organism types collected. We also tested whether the relationship between iAs and d^15^N differed between the lakes by including an interaction term in a modification of linear regression. Next, we compared littoral sediment total As concentrations from 4 to 9 regional lakes (Table 2) to total As in species at multiple trophic levels. We fit different linear regressions to each of six response variables testing the extent that total As in littoral sediments predicts total As concentrations in the organisms. Finally, we tested the relationship between the concentration of total As in littoral sediment against the concentration of iAs (arsenate + arsenite) in five food web constituents, again using linear regression. Regression curve fits are reported as R^2^ values with statistical significance reported as P values.

**Table 2:** Mean total As concentrations ±1 standard deviation in sediment (μg As g^-1^), water (μg As L^-1^), and organisms (μg As g^-1^) in study lakes.

Mean μg As g^-1^	Angle	Arbor	Bonney	Dolloff	Killarney	Mirror	Pine	Spider	Steel	Trout	R^2^	p-value
littoral sediment	35 ± 3.8	12	10 ± 0.3	20	213 ± 84	19	9	22	50 ± 27	7		
oxic water (μg As L^-1^)	1.5 ± 0.1		0.03 ± 0.04		20 ± 4.4				1.8 ± 0.4		0.99	0.00598
periphyton	305 ± 15	17 ± 0.4	16 ± 14	81 ± 23	627 ± 92	156 ± 26			128 ± 6.7	25 ± 3.2	0.86	0.000904
phytoplankton	65 ± 42	6.9 ± 1.0	7.3 ± 4.6	11 ± 3.9	392 ± 294	27 ± 2.0		40 ± 32	61 ± 68	6.7 ± 1.8	0.99	1.51e-8
macrophyte	50 ± 28				273 ± 59							
chironomid	5.7 ± 0.5				27 ± 3.1							
snail	17 ± 11		2.9 ± 0.4	3.5 ± 1.3	45 ± 15				15 ± 14	3.7 ± 0.8	0.95	0.00114
zooplankton	6.4 ± 4.7		1.0 ± 0.6		14 ± 6.8				4.7 ± 3.7		0.91	0.0469
fish	0.30 ± 0.14		0.07 ± 0.08		0.63 ± 0.20		0.17 ± 0.08		0.44 ± 0.08		0.78	0.0479

## Results and discussion

### Arsenic in littoral sediments and surface waters

Total arsenic concentrations in littoral sediments in the ten study lakes ranged from 7 to over 200 mg kg^-1^ (Table 1). This study focuses on shallow lakes, with 8 of the 10 lakes having maximum depths less than 8 m, while Angle Lake and Pine Lake, the two deeper lakes, are included to establish whether arsenic trophic transfer can be universalized across lakes with different depths and mixing regimes. Lake depth and resultant mixing regime play an important role in arsenic distribution. It has been previously documented that arsenic concentrations in the littoral sediments of shallow Lake Killarney are comparable to profundal sediment concentrations (213 and 206 μg As g^-1^, respectively), whereas in deep Angle Lake littoral sediment arsenic concentrations are nearly 6 times less than those in the profundal zone (35 and 208 μg As g^-1^, respectively; [27]). Both lakes thermally stratify in the summer, causing the bottom waters to become at least periodically anoxic [29, 31]. Under anoxic conditions, arsenic bound to iron-containing solids in lakebed sediments is mobilized by reductive dissolution and diffusion into bottom waters [30]. In Angle Lake, thermal stratification is maintained throughout the season, but in Lake Killarney, frequent convective mixing events temporarily break down the thermocline [29, 31]. Previous research has provided evidence of an enhanced renewal of arsenic from profundal sediments to littoral zones in shallow lakes, compared to deeper lakes where steeper morphometry and strong seasonal stratification may limit redistribution of arsenic from deep lake sediments to shallow waters [30–32]. The correlation between arsenic in littoral sediments measured in this study and the summertime oxic water column (measured by [27]) was quite strong (R^2^ = 0.99, P = 0.00598; n = 4; Table 2), while there was no relationship between mean arsenic concentrations in the oxic water column and profundal sediments of these lakes [27]. Although in most deeper lakes maximum contaminant accumulation will occur in the deepest area of lake basins due to sediment focusing [32, 46], metal concentrations in profundal sediments may not be representative of the potential human health risk.

### Food source and the bioavailability of arsenic

Food sources of sunfish and mystery snails (*B*. *chinensis*) are of particular interest because these fish species are known to be harvested for human consumption, and different food sources take up varying levels of arsenic relative to trophic position, feeding behavior, and habitat [27]. Although both *Lepomis gibbosus* and *L*. *macrochirus* (the resident sunfish species in Angle and Killarney Lakes, respectively) have broadly similar diets consisting of a combination of micro- and macroinvertebrates [47], differences in mouth morphology allow them to specialize on different prey [48–51]. *L*. *gibbosus* are considered more molluscivorous because their strong molar-shaped pharyngeal teeth allow them to crush snail shells, whereas *L*. *macrochirus* have small, sharp teeth that support feeding on zooplankton and insect larvae [52]. Andraso [48] also reported mollusks (dreissenids) as the most important type of prey of pumpkinseed diet, whereas bluegill demonstrated a feeding preference for zooplankton and insect larvae. Although most studies suggest sunfish use vegetation primarily for shelter and foraging for insect larvae [49, 50], a study by Taguchi et al. [51] found that the diet of bluegill in a tributary of Lake Biwa, Japan consisted mainly of submerged macrophytes, along with small mollusks and *Chironomidae* larvae.

**Fig 2:**
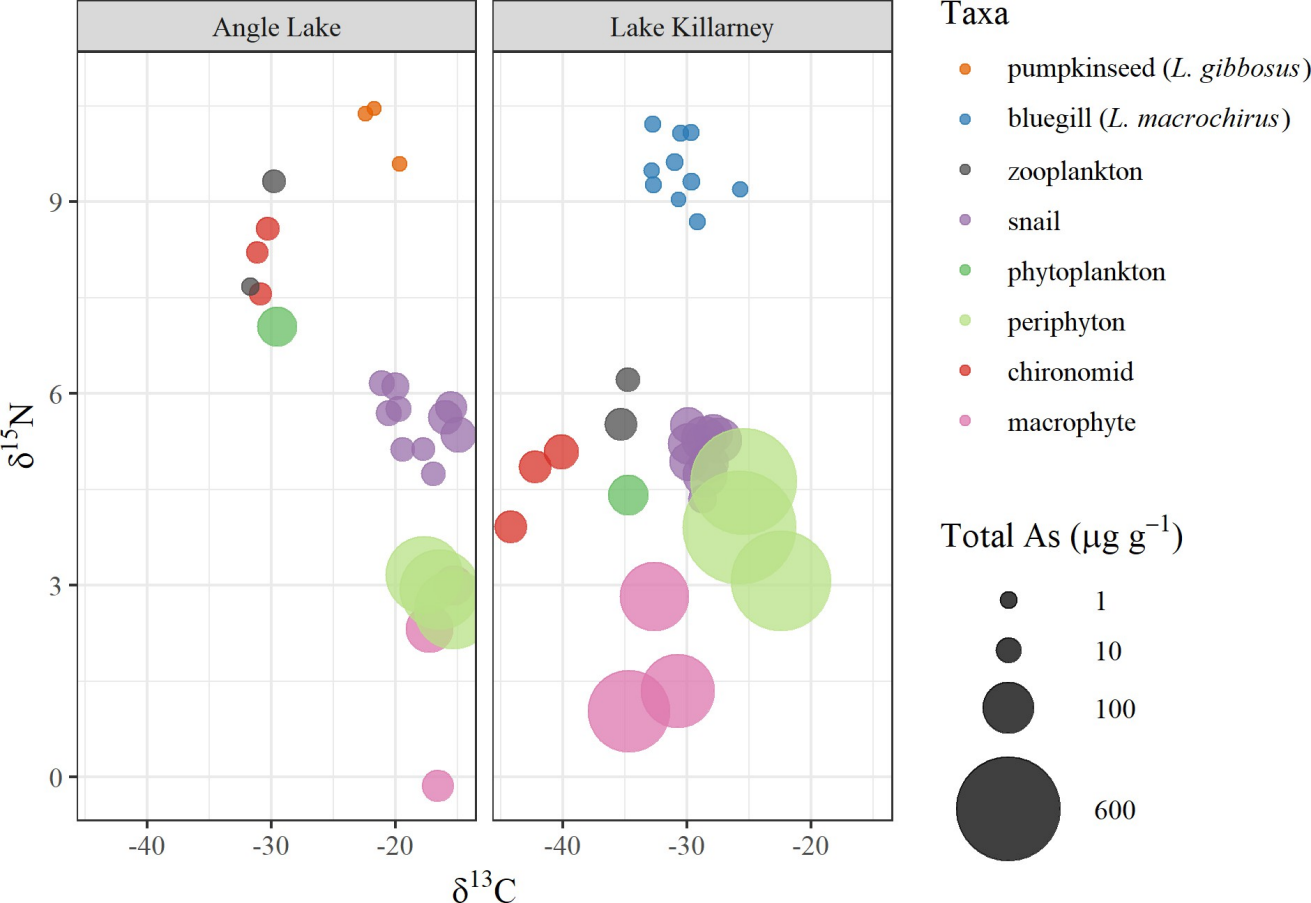
Biplot of δ^13^C versus δ ^15^N and total arsenic concentrations (μg g^-1^) in organisms.

Our results from stable isotope analysis support a varied diet for both sunfish species. Mean δ^13^C values for pumpkinseed sunfish in Angle Lake suggest a diet consisting of a combination of snails, zooplankton, and aquatic insect larvae, while δ^13^C values for bluegill in Lake Killarney support a diet of zooplankton, snails, and macrophytes (Fig 2). Pathways responsible for trophic transfer of arsenic to *B*. *chinensis* snails may vary over time due to known shifts in feeding behavior associated with life stage and resource availability [53]. Laboratory studies reported that juvenile *B*. *chinensis* graze exclusively on periphyton, but individuals switch to both grazing and filter-feeding (phytoplankton) later in life if periphyton availability is limited. Angle Lake δ^13^C values for periphyton, macrophytes, and *B*. *chinensis* were similar and about 12–14 ‰ higher than δ^13^C values for phytoplankton, suggesting that *B*. *chinensis* in Angle Lake primarily graze periphyton and macrophytes (Fig 2; S2 Table). Average δ^13^C values for phytoplankton, periphyton, macrophytes, and *B*. *chinensis* exhibited a similar pattern in Lake Killarney, although a smaller difference between phytoplankton and periphyton was observed.

Periphyton’s tendency to accumulate much higher concentrations of arsenic compared to other primary producers (phytoplankton and macrophytes) may explain why *B*. *chinensis* contained higher concentrations of As compared other primary consumers (zooplankton and *Chironomidae*; Table 2). Average δ^13^C values in zooplankton and *Chironomidae* larvae suggest a stronger dependence on phytoplankton than periphyton as a food source for both of these consumers in Angle and Killarney, resulting in less arsenic exposure and trophic transfer through dietary intake for zooplankton and insect larvae compared to snails.

### Trophic positioning and potential health risk

Both food source and trophic position are important factors in predicting a species’ diet-based arsenic exposure and the subsequent potential health risk from human consumption. Prior work calculated that the arsenic-based human health risk from consumption of *B*. *chinensis* harvested from Lake Killarney was greater than 4 additional cancer occurrences per 1,000 people (99^th^ percentile consumption rate representing subsistence fishers; [27]); much higher than sunfish (1.13 x 10^−5^; [27]) which have more varied diets and higher trophic positioning (Fig 2).

**Fig 3:**
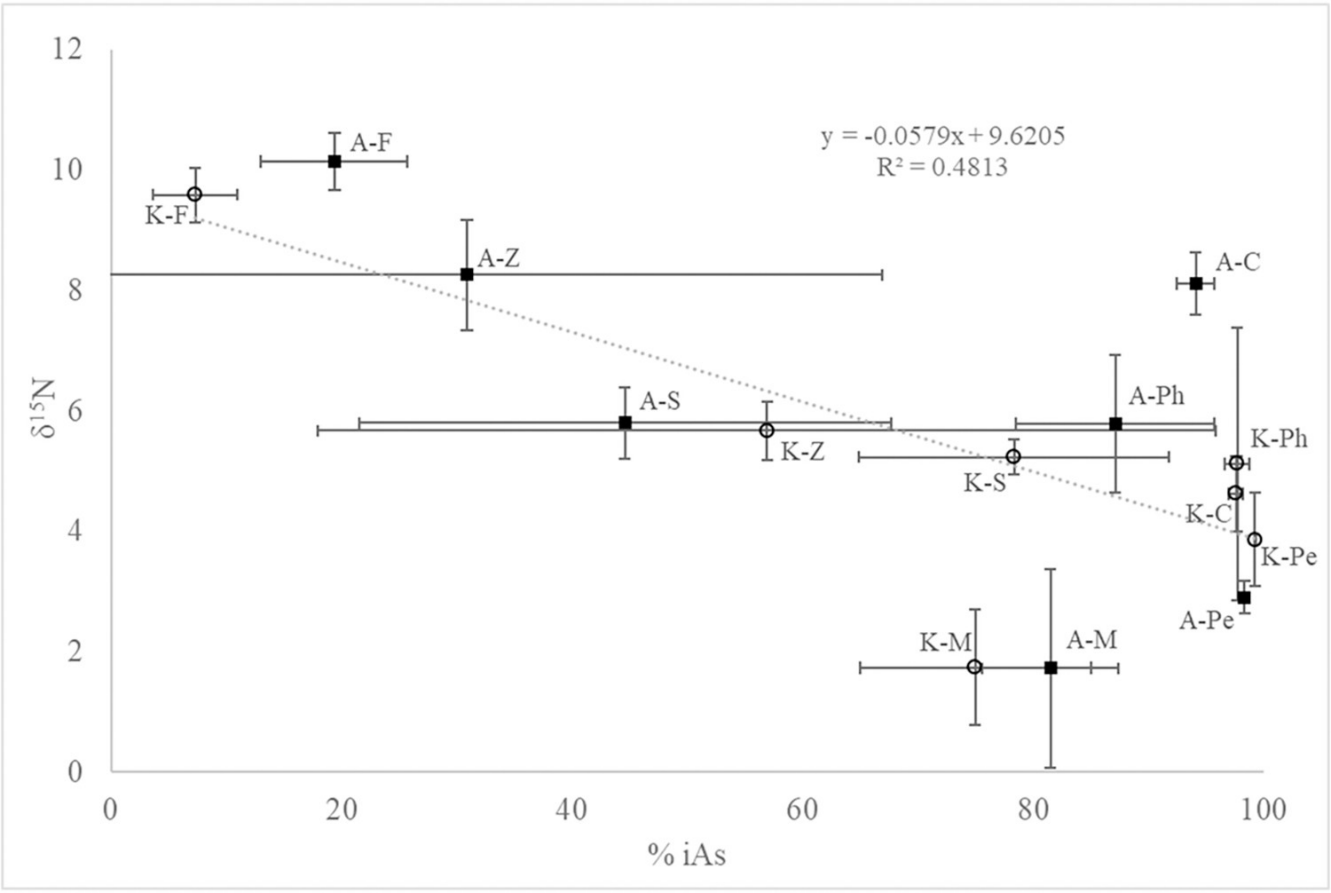
Mean percent inorganic arsenic compared to mean δ^15^N in organisms from Angle Lake and Lake Killarney. Data labels indicate the Lake (A = Angle; K = Killarney) followed by organism type: F = sunfish; Z = zooplankton; S = *B*. *chinensis*; C = *Chironomidae*; M = macrophyte; Ph = phytoplankton; Pe = periphyton.

Human health risk is calculated using only inorganic arsenic species (iAs; [27]). In this study, the fraction of iAs as a percentage of total As in food web components from Lake Killarney and Angle Lake was inversely related to trophic position (δ^15^N) (R^2^ = 0.438, p = 0.0059; Fig 3). At the base of the food web, phytoplankton from Killarney and Angle Lakes contained 98% and 87% iAs, respectively, and periphyton contained almost exclusively iAs (>98%). Interestingly, iAs also comprised a very high percentage of total As in *Chironomidae* with an average of 98% in Killarney and 94% in Angle. This result may be due to their diet including direct consumption of sediment particles [54].

Zooplankton and *B*. *chinensis* contained intermediate iAs fractions, while sunfish, at the highest trophic level, had the lowest iAs fraction, measuring, on average, 7% iAs in *L*. *macrochirus* from Killarney and 19% in *L*. *gibbosus* from Angle (Fig 3). Although both species of sunfish occupy the same trophic position in their respective food webs, the >10% difference in % iAs between *L*. *gibbosus* and *L*. *macrochirus* may reflect key differences in diet. *Lepomis gibbosus* and other molluscivorous predators may have a greater fraction iAs as their diet includes feeding on sediment-dwelling prey, possibly resulting in the inadvertent consumption of iAs in sediment, whereas zooplanktivorous *L*. *macrochirus* would be less likely to be exposed to sediment As while feeding.

Importantly, the relationship between organism trophic position (δ^15^N) and % iAs was not different between Angle and Killarney. The interaction term added to the linear regression was near zero and not statistically significant, indicating that we can fit the same slope within each lake. While additional data from other lakes and members of the food web would increase the ability to make more robust inferences, this result suggests that regardless of physical lake characteristics (depth and mixing regime), trophic positioning can be used to estimate the percentage of iAs in a species, providing an empirical method for predicting consumption-based human health risk.

### Littoral sediment as predictor for total arsenic in organisms

Being able to estimate the proportion of iAs in organisms is only useful in predicting the degree of risk if the magnitude of total As is known. In general, total arsenic concentrations in organisms collected from Angle, Killarney, and 8 other regional lakes decreased as trophic position increased (Table 2). Primary producers accumulated the highest levels of arsenic compared to higher trophic levels. Periphyton arsenic concentrations were the highest of the three types of primary producers (periphyton, phytoplankton, and macrophytes) in all sampled lakes (n = 8), with mean values ranging from 16.4 to 627 μg As g^-1^ in Bonney and Killarney Lakes, respectively. *B*. *chinensis* accumulated the highest As concentrations of the primary consumers in the lakes where they were present (n = 6). Sunfish muscle tissue had the lowest concentrations of As of all organisms collected, ranging from 0.07 μg As g^-1^ (Bonney Lake; *L*. *gibbosus* and *L*. *macrochirus*) to 0.63 μg As g^-1^ (Lake Killarney, *L*. *macrochirus*).

Arsenic concentrations in lake surface waters and across the types of organisms sampled reflected the amount of As in littoral sediment in each lake (Table 2). The correlation between arsenic in littoral sediments and the summertime oxic water column (measured in [27]) was quite strong (R^2^ = 0.982, P = 0.00598; n = 4; Table 2), while there was no relationship between mean arsenic concentrations in the oxic water column and profundal sediments of these lakes [27]. Although in most deeper lakes maximum contaminant accumulation will occur in the deepest area of lake basins due to sediment focusing [32, 46], metal concentrations in profundal sediments may not be representative of the potential human health risk. The concentration of total As in oxic water and organisms collected from at least 4 lakes were regressed with total As concentrations in littoral sediment. Total arsenic in littoral sediment shows strong positive correlation with total arsenic accumulated in phytoplankton, periphyton, zooplankton, *B*. *chinensis*, and sunfish (R^2^ ≥ 0.704, P < 0.05; [27], and this study; Table 2). The strong correlations suggest littoral sediment As concentrations play a role in the extent of As bioavailability. Total arsenic concentrations in littoral sediment are also season-independent, compared to water column arsenic concentrations which can display significant seasonal variation [29].

### Littoral sediment and inorganic arsenic in organisms

**Fig 4:**
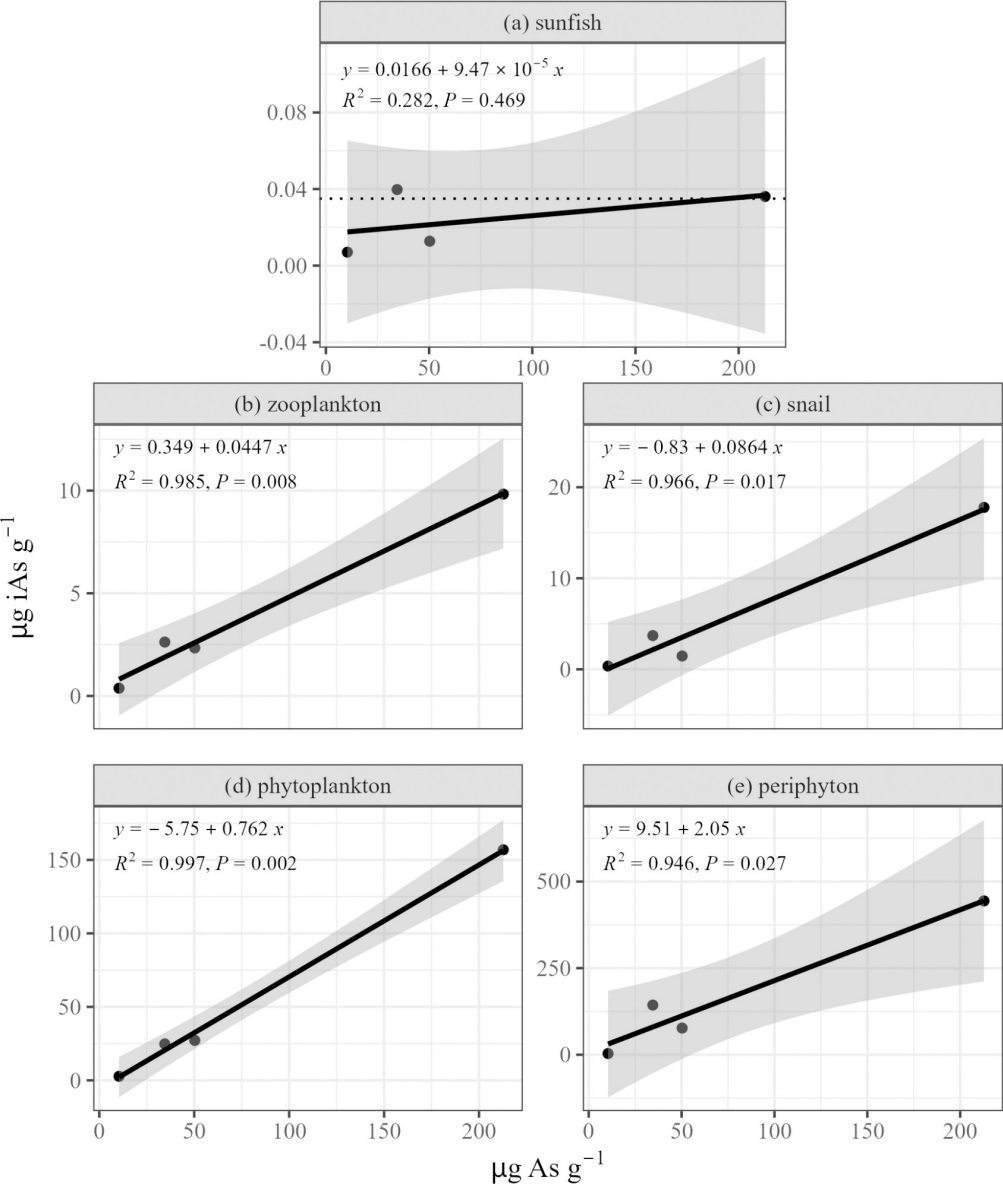
The relationships between total As in littoral sediment (μg As g^-1^) and measured iAs (μg iAs g^-1^) in (a) sunfish, (b) zooplankton, (c) snails, (d) phytoplankton, and (e) periphyton from Angle, Bonney, Killarney, and Steel Lakes. In panel (a), the dotted line represents the threshold for the concentration of iAs in fish required to exceed a 10^−5^ cancer risk at the 99^th^ percentile consumption rate.

To predict the potential degree of risk to humans from consumption of aquatic species in arsenic-contaminated lakes it is necessary to be able to estimate the concentration of inorganic arsenic in edible tissues. We found strong (R^2^ ≥ 0.92) and statistically significant (p < 0.05) relationships between the concentration of As in littoral sediment and iAs in periphyton, phytoplankton, zooplankton, and snails (Fig 4). The relationship between littoral sediment and sunfish was much weaker and not significant, which may result from including two species of sunfish with different feeding behaviors and diets. Angle and Steel Lakes contain *L*. *gibbosus*, whereas the resident sunfish species in Killarney is *L*. *macrochirus*. Bonney Lake hosts populations of both *L*. *gibbosus and L*. *macrochirus*. In a previous study, the difference in total As accumulation between the two sunfish species was determined to be insignificant in Bonney Lake [27], and in this study the relationship between total As and littoral sediment was significant across species (Table 2). Therefore, it is possible that species-specific feeding behaviors may be more important for the concentration of iAs that is accumulated than initially assumed.

While more data is needed to fine tune these empirical relationships and it may be necessary to separate fish species, we can use the equations from Fig 4 to estimate the minimum concentration of total As in littoral sediment that may trigger a potential human health risk from consumption of Chinese mystery snails. Using the risk assessment method as detailed in Hull et al. [27], the littoral sediment As concentration threshold for consuming *B*. *chinensis* at the 90^th^ percentile consumption rate (17.5 g day^-1^; [55]) is 17.66 μg As g^-1^ dry weight sediment. Therefore, based on total As concentrations in littoral sediment in lakes from this study, *B*. *chinensis* from Angle, Killarney, Steel, and Dolloff Lakes (*B*. *chinensis* was not found in Mirror and Spider Lakes) may pose a human health risk if consumed at that rate, but *B*. *chinensis* from the other lakes sampled likely do not (Fig 4).

## Conclusions

This study demonstrates the impact of shallow lake depth and the accompanying polymictic mixing regime on the redistribution of As in deep sediments to the littoral zones of lakes, and ultimately on human health risk from the consumption of edible aquatic species. An in-depth investigation of As speciation and trophic transfer in organisms from shallow Lake Killarney and deep Angle Lake revealed that the association between δ^15^N and % iAs was consistent between lakes that differ greatly in littoral sediment As concentrations, depth, and mixing regime. Furthermore, the strong correlations between total As in littoral sediments and in primary producers and primary and secondary consumers across sampled lakes supports a generalizable proportional trophic transfer of As into tissues of species at higher trophic levels. Although further data collection from a larger number of lakes is needed to create a more robust empirical relationship, especially for fish species with differing feeding behaviors, our results suggest that potential for human health risk from consumption of aquatic species in lakes can be screened for using just total As concentrations in littoral sediments and a knowledge of trophic position of edible aquatic species.

Traditionally, the concentration of heavy metals in lake sediments is determined by sampling from the profundal zone at the deepest point in a lake. This study provides strong evidence of linkages between littoral sediments and the bioavailability of As in lake food webs. Because of the lack of comparable published data, we were not able to perform a meta-analysis to test the significance of the empirical relationships derived in this study with a larger sample size from the literature. Furthermore, models for organisms at higher trophic levels should be species-specific, as became apparent when attempting to correlate littoral sediment with *L*. *macrochirus* and *L*. *gibbosus* grouped as sunfish. Nevertheless, this research suggests measuring As in littoral sediment is an accessible method for assessing whether a lake is at risk of increased arsenic bioavailability. Sampling littoral sediment requires very little equipment, and rapid tests for total As are becoming increasingly accurate and affordable. In conclusion, this research provides the framework for a model that can be used by natural resource managers as a screening tool to assess whether lakes may be vulnerable to posing an arsenic-related human health risk from consumption of aquatic species, warranting further investigation.

## Supporting information

S1 Table Sample size of analyses performed on each type of organism per lake. (PDF)

S2 Table Mean δ^13^C, δ^15^N, and percent inorganic arsenic in food web constituents from Angle Lake and Lake Killarney. (PDF)
