# Supplementary material for: Littoral sediment arsenic concentrations predict arsenic trophic transfer and human health risk in contaminated lakes
Source: PLoS One. 2023 Oct 19;18(10):e0293214. doi: 10.1371/journal.pone.0293214 (PMC10586660; doi:10.1371/journal.pone.0293214)
Supplement: S1 Table — (PDF) [file pone.0293214.s001.pdf]

S1 Table. Sample size of analyses performed on each type of organism per lake.

		**periphyton**			**phytoplankton**			**macrophyte**			**zooplankton**			***Chironomidae***			***B. chinensis***			***L. gibbosus/ L. macrochirus***		
	analyses	*TA*	*AS*	*SI*	*TA*	*AS*	*SI*	*TA*	*AS*	*SI*	*TA*	*AS*	*SI*	*TA*	*AS*	*SI*	*TA*	*AS*	*SI*	*TA*	*AS*	*SI*
Lake	**Angle**	3	3	3	10	4	3	3	3	3	8	3	3	3	3	3	20	3	10	15	3	10
	**Arbor**	3			2																	
	**Bonney**	3	3		2	2					2	2					7	3		12	3	
	**Dolloff**	3			2												7					
	**Killarney**	3	3	3	10	3	3	3	3	3	8	3	3	3	3	3	20	3	10	20	3	10
	**Mirror**	3			2																	
	**Pine**																			12		
	**Spider**				2																	
	**Steel**	3	3		2	2					2	2					7	3		10	3	
	**Trout**	3			2												9					

[TA] total arsenic
[AS] arsenic speciation
[SI] stable isotope analysis
