# Supplementary material for: Littoral sediment arsenic concentrations predict arsenic trophic transfer and human health risk in contaminated lakes
Source: PLoS One. 2023 Oct 19;18(10):e0293214. doi: 10.1371/journal.pone.0293214 (PMC10586660; doi:10.1371/journal.pone.0293214)
Supplement: S2 Table — (PDF) [file pone.0293214.s002.pdf]

S2 Table. Mean $\delta^{13}C$, $\delta^{15}N$, and percent inorganic arsenic in food web constituents from Angle Lake and Lake Killarney.

		stable isotope			speciation	
**Lake**	**organism**	**$\delta^{13}C$**	**$\delta^{15}N$**	**n**	**%iAs**	**n**
Angle	sunfish	-21.03 ± 1.00	10.22 ± 0.39	10	19.4 ± 6.4	3
Killarney	sunfish	30.48 ± 2.15	9.50 ± 0.50	10	7.3 ± 3.7	3
Angle	zooplankton	-30.14 ± 1.10	8.26 ± 0.92	3	30.9 ± 36.0	3
Killarney	zooplankton	-34.23 ± 1.46	5.67 ± 0.49	3	56.9 ± 39.0	3
Angle	*Bellamya*	-18.22 ± 2.25	5.55 ± 0.45	10	44.6 ± 23.1	3
Killarney	*Bellamya*	-28.80 ± 0.86	5.09 ± 0.35	10	78.4 ± 13.4	3
Angle	Chironomidae	-30.77 ± 0.42	8.11 ± 0.51	3	94.2 ± 1.6	3
Killarney	Chironomidae	-42.19 ± 2.04	4.62 ± 0.62	3	97.7 ± 0.6	3
Angle	Macrophyte	-16.41 ± 0.96	1.73 ± 1.65	3	81.5 ± 5.9	3
Killarney	Macrophyte	-32.70 ± 1.96	1.74 ± 0.96	3	75.0 ± 10.0	3
Angle	phytoplankton	-29.81 ± 0.72	5.79 ± 1.14	3	87.2 ± 8.6	4
Killarney	phytoplankton	-35.14 ± 3.61	5.12 ± 2.26	3	97.7 ± 1.0	3
Angle	periphyton	-16.52 ± 1.18	2.91 ± 0.27	3	98.4 ± 0.04	3
Killarney	periphyton	-24.57 ± 1.84	3.87 ± 0.78	3	99.3 ± 0.1	3
